# A rare case of a cholecysto-duodenocolonic fistula secondary to cholelithiasis

**DOI:** 10.1093/jscr/rjae175

**Published:** 2024-03-22

**Authors:** Tejminder S Sidhu, Shaurya Jhamb, Matan M Ben David

**Affiliations:** College of Medicine and Dentistry, James Cook University, Queensland 4814, Australia; Department of Surgery, Townsville University Hospital, Townsville 4814, Australia; College of Medicine and Dentistry, James Cook University, Queensland 4814, Australia; Department of Surgery, Townsville University Hospital, Townsville 4814, Australia; Department of Surgery, Townsville University Hospital, Townsville 4814, Australia

**Keywords:** cholelithiasis, internal biliary fistula, cholecysto-duodenocolonic fistula, cholecystoenteric fistula

## Abstract

Internal biliary fistula is a rare but well-known complication of cholelithiasis. It is a notoriously challenging entity to diagnose and manage. Gallstones are often the causative factor in the formation of a cholecystoenteric fistula, with the most common internal biliary fistula being a cholecystoduodenal fistula followed by a cholecystocolonic fistula. Rarely, do these fistulae exist simultaneously. Here, we present an uncommon case of cholecysto-duodenocolonic fistula.

## Introduction

Spontaneous internal biliary fistula or cholecystoenteric fistula is an uncommon complication of gallstone disease. Prevalence of cholecystoenteric fistula is thought to be between 0.4% and 1.9% of patients with cholelithiasis [1, 2]. Although the common culprit of this condition is cholelithiasis, other causes including malignancy, diverticulitis, trauma inflammatory bowel disease, and iatrogenic injury should also be considered [3]. Generally, chronic inflammation of the gallbladder secondary to cholelithiasis is responsible for the formation of this type of fistula. The most common internal biliary fistula is a cholecystoduodenal fistula, followed by cholecystocolonic fistula with others being significantly more rare. Patients with cholecystoenteric fistulae often present with a vague constellation of abdominal symptoms ranging from asymptomatic to chronic diarrhoea and abdominal pain but may also include fever, jaundice, nausea, vomiting, weight loss, or bowel obstruction (gallstone ileus). Diagnosis is often challenging due to the vague nature of the presenting symptoms and in some cases is discovered intraoperatively. While a single cholecystoenteric fistula is uncommon, the presence of a second fistula is significantly more remarkable. Here, we present an uncommon case of a cholecysto-duodenocolonic fistula in a patient with cholelithiasis.

## Case presentation

An 83-year-old Gentleman presented to the emergency department (ED) with vague epigastric and right upper quadrant pain on a background of chronic diarrhoea. Examination indicated mild diffuse tenderness and Murphy’s sign was negative with no further localizing signs. The patient’s past medical history included coronary artery bypass graft for ischaemic heart disease, hypercholesterolaemia, and hypertension. The patient was in otherwise relatively good health and despite his age was independent with activities of daily living. Initial bloods were unremarkable, and contrast-enhanced computed tomography on initial presentation demonstrated diffuse gastroenteritis; therefore, he was discharged from ED. On second presentation, the patient was admitted, and initial management was supportive with intravenous (IV) rehydration and analgesia. The patient remained stable, however, had ongoing symptoms of diarrhoea, hence a colonoscopy and gastroscopy were completed. The patient denied any previous abdominal surgery; however, on colonoscopy, a fistula between the colon and the small bowel was demonstrated at the hepatic flexure. The gastroenterologist was puzzled as there were no previous abdominal surgeries.

**Figure 1:**
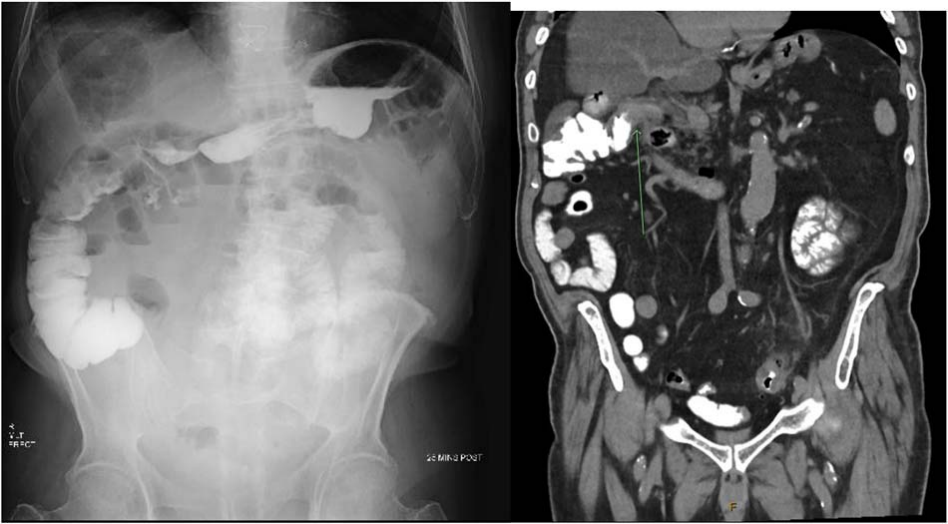
X-ray post barium swallow demonstrating early contrast opacification of the ascending colon with coronal slice of CT confirming the presence of a fistula between the duodenum and ascending colon below the hepatic flexure.

**Figure 2:**
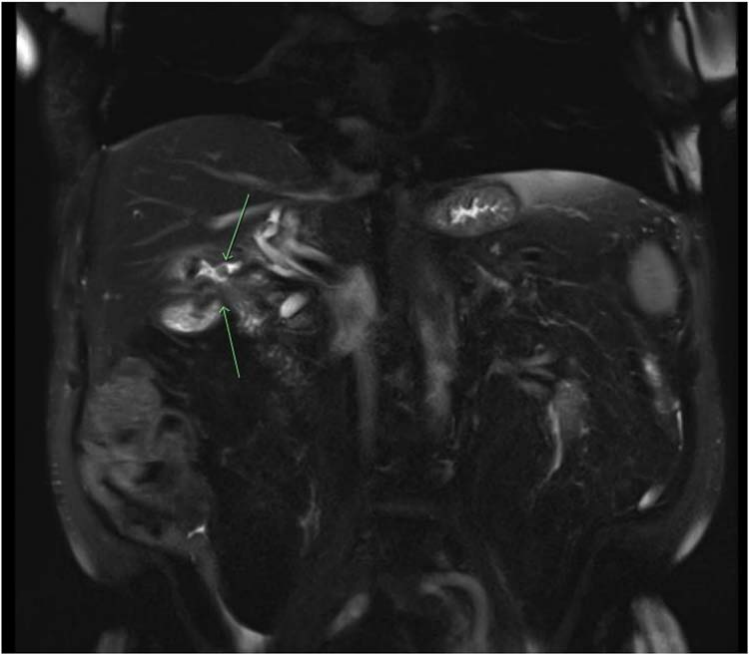
Coronal slice of a magnetic resonance cholangiopancreatography (MRCP) with IV gadolinium demonstrating an abnormal fistulous connection between the contracted small calibre gallbladder and the colon just below the hepatic flexure; there is evidence of a second fistulous tract between the gallbladder and the duodenum.

Consequently, a barium swallow and MRI abdomen were completed demonstrating the fistula between the first part of the duodenum and the ascending colon below the hepatic flexure (Figs 1 and 2). Surgical opinion and management of the patient was then sought. A subtotal cholecystectomy, partial duodenectomy, and partial resection of the colon was completed to excise the fistulous tracts. Given the patients age, he was sent to the intensive care unit for further monitoring. Day 3 postoperatively, the patient had ongoing pain and developed new tachycardia, and CT at this time demonstrated an obstruction at the colonic suture line. The patient was taken back to the operating room and a completion right hemicolectomy was performed. Histopathology from the original operation demonstrated heavy mucosal inflammation with a fistula identified histologically. The patient recovered relatively well and was able to be discharged home. The patient remained healthy and continued to be living independently in the community at 6-month follow-up.

## Discussion

Cholecystoenteric fistula is an abnormal connection that forms between the gallbladder and the bowel usually secondary to cholelithiasis. This is an uncommon entity and presents an interesting diagnostic and management challenge. The most common of the internal biliary fistulae is the cholecystoduodenal fistula which comprise between 60% and 80% of all cases, with cholecystocolonic fistula present in about 15% of cases [4]. Internal biliary fistulae are usually found in elderly co-morbid patients with higher rates in females. The pathophysiologic basis of this condition is chronic inflammation of the gallbladder secondary to cholelithiasis causing ischaemia followed by necrosis of the gallbladder wall [5]. Consequently, the gallstone erodes through the gallbladder wall into an adjacent organ causing an internal biliary fistula [5].

The management of cholecystoenteric fistulae should be completed by a Surgeon with expertise in hepatobiliary reconstruction. Historically, patients have been managed with open cholecystectomy with or without bile duct exploration, excision of the fistula tract, and repair of the penetrated portion of bowel [6]. Endoscopic management options have a role in management particularly in cases of Bouveret syndrome or gallstone ileus [7, 8]. Laparoscopic techniques have also been found to safe and reliable options in surgery for cholecystoenteric fistulae, with one study finding no difference in complication rates between laparoscopic and open options [9]. However, in cases with extensive inflammation and abnormal anatomy such as in this case, open techniques are preferred.

## Conclusion

Cholecystoduodenal and cholecystocolonic fistulae are the most common of the internal biliary fistulae; however, they are rarely found simultaneously. This case is a rare example of a cholecysto-duodenocolonic fistula managed with open subtotal cholecystectomy and partial resection of the duodenum and colon.
